# Target of Rapamycin Complex 2 regulates cell growth via Myc in *Drosophila*

**DOI:** 10.1038/srep10339

**Published:** 2015-05-22

**Authors:** Ying Kuo, Huanwei Huang, Tao Cai, Tao Wang

**Affiliations:** 1 National Institute of Biological Sciences, Beijing, China

## Abstract

Target of rapamycin (TOR) is an evolutionarily conserved serine/threonine protein kinase that functions as a central regulator of cellular growth and metabolism by forming two distinct complexes: TOR complex 1 (TORC1) and TORC2. As well as TORC1, TORC2 plays a key role in regulation of cell growth. But little is known about how TORC2 regulates cell growth. The transcription factor Myc also plays a critical role in cell proliferation and growth. Here we report that TORC2 and Myc regulate cell growth via a common pathway. Expression of Myc fully rescued growth defects associated with *lst8* and *rictor* mutations, both of which encode essential components of TORC2. Furthermore, loss of TORC2 disrupted the nuclear localization of Myc, and inhibited Myc-dependent transcription. Together, our results reveal a Myc-dependent pathway by which TORC2 regulates cell growth.

The Target of rapamycin (TOR) signaling pathway consists of a set of biochemical processes, which in response to environmental cues and growth factors regulates organismal and cellular growth. TOR is an evolutionarily conserved serine/threonine protein kinase and functions as a core catalytic component of two distinct multiprotein complexes, TOR complex 1 (TORC1) and TORC2^1^,^2^,^3^. TORC1 controls cell autonomous growth in response to nutrient availability and growth factors, whereas mTORC2 mediates cell proliferation and cell survival by activating several kinases within the AGC family, including AKT, serum and glucocorticoid-regulated kinase (SGK), and protein kinase C (PKC)^4^,^5^.

Although the signaling networks regulated by TORC2 are not completely understood, studies in mice, *Drosophila*, and *Caenorhabditis elegans* have demonstrated a role for TORC2 in cell growth^6^,^7^,^8^,^9^. Thus, TORC2 is emerging as a pivotal player in many cancers^10^,^11^. As a downstream target of TORC2, AKT plays essential roles in several important cellular processes, including growth, proliferation, survival, and metabolism^12^. Studies in multiple systems have demonstrated that TORC2 inactivates FOXO (forkhead transcription factors of the O-class) through AKT signaling, but it is clear that AKT and FOXO do not mediate TORC2-regulated cell growth^6^,^7^,^9^,^13^. It is therefore critical to identify signaling pathways that act downstream of TORC2 to regulate cell growth.

The *myc* family of proto-oncogenes encode the transcription factors C-myc, N-myc, and L-myc^14^. Myc proteins play pivotal roles in cell growth and proliferation through the transcriptional regulation of a large number of target genes. As such, dysregulation of Myc contributes to the genesis of many human cancers^14^,^15^. *Drosophila* has a single *myc* gene (*dmyc*), which plays a key role in controlling cell size and growth rates by regulating the transcription of mRNAs, ribosomal RNA, and small noncoding RNAs^16^,^17^. Hypomorphic *dmyc* mutations reduce the rate of growth and final size of animals^18^,^19^,^20^,^21^, and dMyc overexpression results in cells and animals that are larger than normal^22^,^23^,^24^.

Myc has been shown to interact with the TOR pathway. In *Drosophila*, dMyc is an important mediator of TOR-dependent growth and metabolism^25^, and inhibition of TOR leads to the post-transcriptional down-regulation of dMyc^16^,^17^. In mammalian cells, a large-scale quantitative phosphoproteomics study has shown that TOR phosphorylates C-Myc at Ser77^26^. Moreover, similar hypogrowth phenotypes are seen in *dmyc*, *lst8*, and *rictor* mutant animals (the latter two encode essential components of TORC2), suggesting that Myc is an essential link between TORC2 and cell growth^7^,^9^,^23^.

Using the *Drosophila* model system, we found that both cellular and organismal growth defects of *dmyc* mutant animals were not exacerbated by the loss of LST8. Ectopic expression of dMyc completely rescued the growth defects of both *lst8* and *rictor* mutant animals, including reduced body weight and shrunken eyes and wings. Moreover, the nuclear localization of dMyc was disrupted in *lst8* or *rictor* mutant cells. Furthermore, gene expression profiling revealed that a large set of growth related genes was dysregulated in *dmyc*, *lst8*, and *rictor* mutant animals. Our findings suggested that Myc functions downstream of TORC2 to regulate cell growth.

## Materials and Methods

### *Drosophila* stocks

*The w*^*1118*^ (wild-type control), *w hs-flp;Act*>*CD2*>*Gal4,UAS-GFP/SM5-TM6B, FM7/l;CyO/Sp, FM7;TM2/TM3, ub-RFP hs-flp FRT19A, lst8*^*1*^ *FRT19A, dm*^*P0*^*FRT19A/CDX, dm*^*P0*^ *lst8*^*1*^ *FRT19A/CDX, lst8*^*1*^ *hs-flp ub-RFP FRT19A, dm*^*P0*^ *hs-flp ub-RFP FRT19A, rictor*^*Δ1*^*, lst8*^*1*^*;en-gal4/CyO, lst8*^*1*^*;GMR-gal4/CyO, lst8*^*1*^ *FRT19A/FM7;Tub-dmyc/TM6B, rictor*^*Δ1*^ *FRT19A/FM7; Tub-dmyc/TM6B and pink1B9* were kept or generated in Dr. T. Wang’s lab. The *yw*; Tub-HA-dmyc flies were obtained from Dr. P. Gallant. The *dm*^*P0*^ and *UAS-dmyc* flies were gifts from Dr. R. Eisenman. All fly stocks were maintained at 22–25 °C on standard medium unless otherwise specified. Heat shock-induced experiments were carried out by shifting flies to 37 °C for 30 mins to 1 hour at the 1^st^ instar lava stage (for discs) or at the 6–8 hour embryo stage for fat body.

### Immunostaining

Fat body tissue from third-instar larvae was dissected, fixed with 8% paraformaldehyde (PFA) dissolved in phosphate-buffered saline, and labeled with antibodies against dMyc (1:50; gift Dr. R. Eisenman), followed by Alexa Fluor 488 goat anti-mouse IgG secondary antibodies (1:1000; Invitrogen), or FITC phalloidin (green) and Hoechst 33342 (1:1000). Images were acquired using a Nikon Eclipse microscope equipped with Nikon DS-Qi1Mc camera.

### Flow cytometric analysis

First instar larvae of *lst8*^1^ *hs-flp ub-RFP FRT19A*/*dm*^*P0*^ *lst8*^1^ *FRT19A*, *dm*^*P0*^ *hs-flp ub-RFP FRT19A*/*dm*^*P0*^ *lst8*^1^ *FRT19A* flies were heat shocked at 37 °C for 1 hour. Approximately 30–40 third-instar larval wing discs were dissected, trypsinized, and stained with Hoechst 33342 (Invitrogen). Analysis was performed with a FACS Vantage (BD Biosciences) and data analyzed with Cell Quest (BD Biosciences) software.

### Scanning electron microscopy (SEM)

SEM was performed as described^9^. Briefly, heads were dissected from newly enclosed male flies reared at 25 °C, dehydrated with a series of ethanol dilutions, and immersed in hexamethyldisilazane. Samples were coated with gold/palladium and mounted after the solvent was evaporated off. The samples were examined with a JEOL JSM- 5800 microscope. The area of 20 ommatidia in high-magnification SEM pictures was measured with ImageJ, and the mean ommatidium size was calculated.

### Phosphate affinity SDS-PAGE and western blot

Mobility shift of phosphorylated dMyc protein was detected by phosphate affinity SDS-PAGE using acrylamide-pendant phos-tag (Phos-tag AAL-107). Briefly, 50–100 μM phos-tag acrylamide and 100–200 μM MnCl_2_ were added to normal 6% polyacrylamide gel. After electrophoresis, the gel was washed with transfer buffer containing 1 mM EDTA for 10 min with gentle agitation, and then with transfer buffer without EDTA for 10 min. Proteins were transferred to Immobilon-FL transfer membranes (Millipore). For western blotting, pupae were homogenized in SDS sample buffer with a pellet pestle (Kimble-Kontes), and the proteins were fractionated using SDS-PAGE. The proteins were transferred to Immobilon-FL transfer membranes in Tris-glycine buffer. The blots were probed with mouse anti-HA 1:500 (Cell Signaling), mouse anti-dMyc (1:50), and rabbit anti-α tubulin 1:15000 (Sigma), and subsequently with IRDye 800-labeled anti-mouse IgG and IRDye 680-labeled anti-rabbit IgG (Licor). Signals were detected using an Odyssey infrared imaging system.

### RNA-seq analysis

Reads were mapped to the *Drosophila* genome (BDGP5.25) using TopHat (v2.0.10) by allowing up to two mismatches^27^. Expression of genes in the RNA-seq data was measured by calculating reads per kilobase per million mapped reads (RPKM). P-values to detect differential expression were calculated by cufflinks (v2.0.2)^28^. The criteria for differential expressed genes in a mutant were defined as more than two-fold change in RPKM and less than 0.01 in P-values. Gene ontology analysis was performed with DAVID online ( http://david.abcc.ncifcrf.gov/)^29^.

## Results

### Myc and TORC2 function in a linear pathway

**Figure 1:**
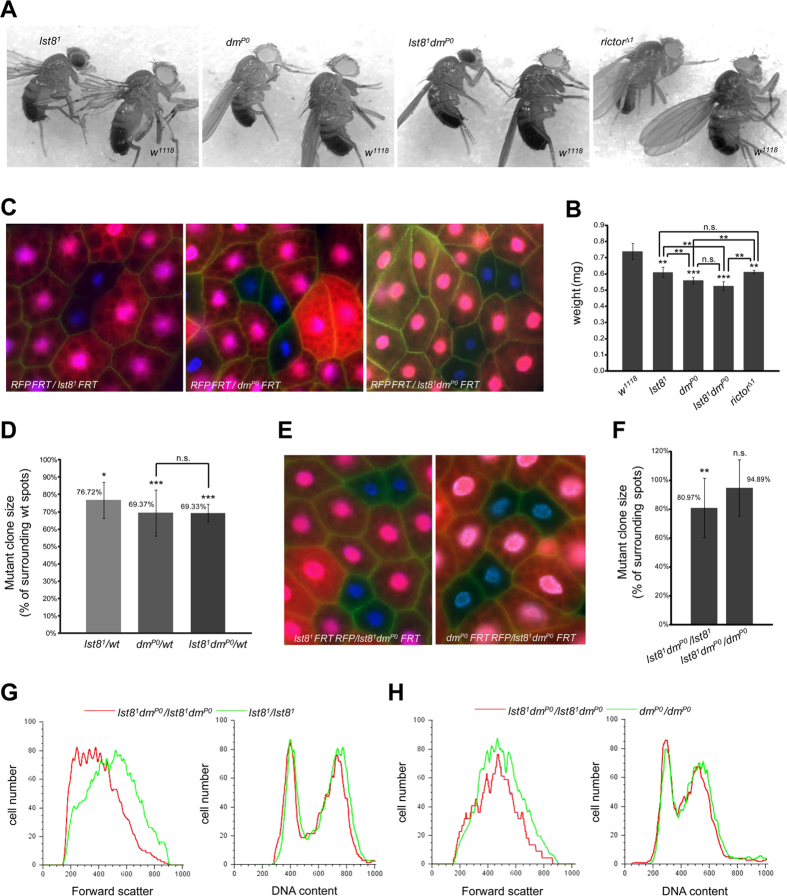
Common pathway for TORC2 and Myc regulated cell growth. **** (**A**) Size comparisons between wild type (*w*^*1118*^) and *lst8*^1^, *dm*^*P0*^, *lst8*^*1*^ *dm*^*P0*^, or *rictor*^*Δ1*^ flies. One-day-old male flies were used for the analysis. (**B**) Quantification of animal weights. Genotypes of one-day-old male flies are shown. Asterisks indicate statistically significant differences (Student’s unpaired t-test, **p < 0.01; ***p < 0.001; n.s., not significant). (**C–F**) Comparisons between *lst8*^*1*^ *dm*^*P0*^, *lst8*^1^, and *dm*^*P0*^ fat body cells. (**C**) The FLP/FRT system was used to generate mosaic *lst8*^1^, *dm*^*P0*^, or *lst8*^*1*^ *dm*^*P0*^ mutant clones marked by the absence of RFP (*hs-flp ub-RFP FRT/lst8*^1^ *FRT*, *hs-flp ub-RFP FRT/dm*^*P0*^ *FRT*, and *hs-flp ub-RFP FRT/lst8*^*1*^ *dm*^*P0*^ *FRT* animals, respectively) (red, RFP; green, phalloidin; blue, DAPI). (**D**) Relative size of *lst8*^1^, *dm*^*P0*^, or *lst8*^*1*^ *dm*^*P0*^ cells compared with controls. Asterisks indicate statistically significant differences from wild-type cells (Student’s unpaired t-test, ***p < 0.001; n.s., not significant). (**E**) The FLP/FRT system was used to generate mosaic *lst8*^*1*^ *dm*^*P0*^ double mutant clones in larval fat bodies of *lst8*^1^ *hs-flp ub-RFP FRT/lst8*^*1*^ *dm*^*P0*^ *FRT* and *dm*^*P0*^ *hs-flp ub-RFP FRT/lst8*^*1*^ *dm*^*P0*^ *FRT* animals, respectively. The *lst8*^*1*^ *dm*^*P0*^ mutant cells are marked by the absence of RFP (red, RFP; green, Phalloidin; blue, DAPI). (**F**) Relative sizes of *lst8*^*1*^ *dm*^*P0*^ double mutant cells compared with *lst8*^1^ or *dm*^*P0*^ cells. Asterisks indicate statistically significant differences from single mutant cells (Student’s unpaired t-test, **p < 0.01; n.s., not significant). (**G** and **H**) Flow cytometry was performed on dissociated wing discs from *lst8*^1^ *hs-flp ub-RFP FRT/lst8*^*1*^ *dm*^*P0*^ *FRT* and *dm*^*P0*^ *hs-flp ub-RFP FRT/lst8*^*1*^ *dm*^*P0*^ *FRT* animals. Cells lacking *lst8* (*lst8*^1^*/lst8*^1^; green trace in G), *dm* (*dm*^*P0*^*/dm*^*P0*^; green trace in H), or *lst8* and *dm* (*lst8*^*1*^ *dm*^*P0*^*/lst8*^*1*^ *dm*^*P0*^; red trace) were compared. Hoechst 33342 staining was used to assess DNA content (right), and FSC was used to quantify cell size (left).

Genetic disruption of TORC2 or Myc cell-autonomously reduces growth rates and cell size, resulting in reduced tissue growth and decreased body weight^9^,^23^. We reasoned that TORC2 and Myc might function in the same pathway to regulate cell growth. To test this hypothesis, we analyzed the body size of 4 mutant fly strains: *lst8*^1^, *dm*^*P0*^, *lst8*^1^ *dm*^*P0*^, and *rictor*^*Δ1*^, which disrupt the *lst8*, *dmyc*, *lst8 dmyc,* and *rictor* genes*, r*espectively. Although reduced body size and body weight were observed in *lst8*^1^, *dm*^*P0*^, and *rictor*^*Δ1*^ flies compared to wild type (*w*^*1118*^), loss of dMyc reduced body weight to a larger extent than loss of *lst8* or *rictor* which both disrupting TORC2 (Fig. 1A,B). Importantly, *lst8*^1^ *dm*^*P0*^ double mutant animals exhibited body weights similar to *dm*^*P0*^ flies, indicating that loss of TORC2 has less impact on growth in TORC2 may be epistatic to Myc in *dm*^*P0*^ mutant.

Using the *hs-FLP*/*FRT* system, we next generated clones of mutant cells in heterozygous fat body tissues. Mutant clones were marked by the absence of red fluorescent protein (RFP) and we assessed larval fat body cell size by staining the tissue with phalloidin to visualize cell boundaries (Fig. 1C). Similar to body weight, loss of LST8, which completely disrupts TORC2 activities, reduced cell size to 76.72% of surrounding control cells, whereas loss of dMyc alone or LST8 and dMyc reduce cell size to 69.37% and 69.33% of control values, respectively (Fig. 1D). To compare growth rates between *lst8*^1^, *dm*^*P0*^, and *lst8*^1^ *dm*^*P0*^ cells in greater detail we used another mosaic system, in which *lst8*^1^ *dm*^*P0*^double mutant clones were generated in a *lst8*^1^ or *dm*^*P0*^single-mutant genetic background (Fig. 1E). Cell size analysis revealed that *lst8*^1^ *dm*^*P0*^ fat body cells were smaller (80.97%) than neighboring *lst8*^*1*^ cells. However, this size reduction was not seen when *lst8*^1^ *dm*^*P0*^ double mutant cells were generated in a *dm*^*P0*^ genetic background (Fig. 1E,F), indicating that loss of dMyc exacerbated growth defects associated with *lst8*^1^, but loss of LST8 did not affect *dm*^*P0*^ cells.

We next generated double mutant *lst8*^1^ *dm*^*P0*^ clones of cells in *lst8*^1^ or *dm*^*P0*^ developing wing discs. Cell size was examined using fluorescence-activated cell sorting (FACS) and forward scatter (FSC) analysis was used to measure cell volumes. This analysis confirmed that loss of dMyc further reduced the size of *lst8*^1^ mutant cells, but the loss of LST8 did not affect *dm*^*P0*^ mutant cells (Fig. 1G,H, left panels). DNA profiles demonstrated that loss of TORC2 and dMyc did not alter cell cycle phasing (Fig. 1G,H, right panels), consistent with previous findings that reductions in cell size associated with the loss of LST8 and dMyc do not result from changes in cell proliferation. These data suggest that dMyc is a potential downstream effector of the TORC2 signaling pathway to mediated cell growth. However, given the essential role of dMyc in organismal growth and development, disrupting TORC2 signaling through the loss of LST8 will likely not abrogate dMyc function completely. This may explain why reductions in cell size were more remarkable in *dm*^*P0*^, and *lst8*^1^ *dm*^*P0*^mutant cells than in *lst8*^1^ cells.

### Myc functions downstream of TORC2 to regulate cell growth

**Figure 2:**
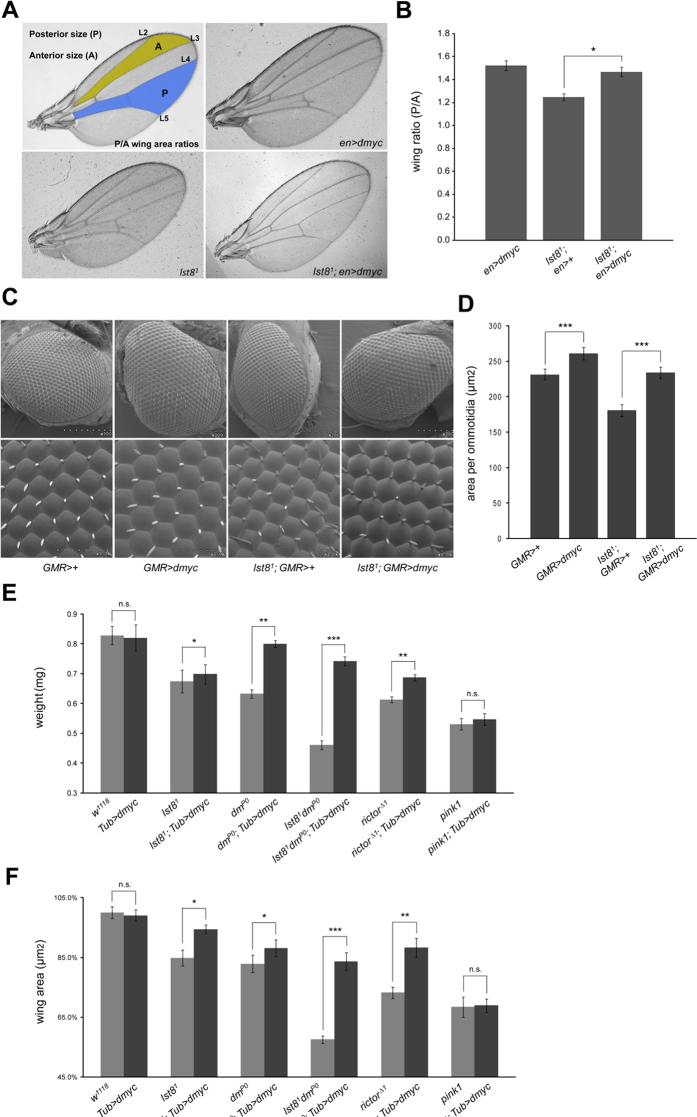
Rescue of TORC2-associated growth defects by dMyc expression. **** (**A**–**B**) Overexpression of dMyc increased the size of the posterior wing compartment in *lst8* mutants. The *en-GAL4* was used to drive *UAS-dmyc* expression in the posterior compartment of the wing (*en>dmyc: en-gal4/UAS-dmyc*). Posterior size (P) and anterior size (**A**) were assessed by measuring the area between vein L4 and L5 (blue, P) and the area between vein L2 and L3 (yellow, A) (**B**) The P/A compartment ratio for *en>dmyc*, *lst8*^1^; *en>+*, and *lst8*^1^; *en>dmyc* wings (15 wings per genotype) are shown. Statistical analysis indicates that dMyc overexpression rescued the *lst8* mutant phenotype. Asterisks indicate statistically significant differences (Student’s unpaired t-test, ***P < 0.001). (**C**–**D**) Overexpression of dMyc increases the size of ommatidia in *lst8* mutants. (**C**) Lateral view of compound eyes from *GMR>dmyc* (*GMR-gal4/UAS-dmyc*), *lst8*^1^; *GMR>+*, and *lst8*^1^; *GMR>dmyc* animals. SEM magnification is 200× or 1000× for upper and lower panels, respectively. (**D**) Ommatidial size for *GMR>dmyc*, *lst8*^1^; *GMR>+*, and *lst8*^1^; *GMR>dmyc* eyes. Five eyes of each genotype were analyzed (Student’s unpaired t-test, ***P < 0.001). (**E**–**F**) The *tub-dmyc* transgene rescued growth defects associated with *lst8*^1^ or *rictor*^*Δ1*^ flies. Body weight (**E**) and wing area (**F**) were analyzed. One hundred one-day-old male flies of each genotype were used to assess body weight, and at least 10 wings per genotype were used to calculate wing area. Asterisks indicate statistically significant differences between flies with the *tub-dmyc* transgene or not (Student’s t-test, **p < 0.01; ***p < 0.001; n.s., not significant).

To determine whether dMyc functions downstream of TORC2 signaling, rescuing experiments were performed in which *engrailed-Gal4* (*en-Gal4*) was used to drive expression of *UAS-dmyc* in the posterior compartment of developing wings in *lst8*^1^ animals. Expression of dMyc in *lst8*^1^ animals rescued cell growth within the posterior compartment without affecting the anterior compartment (Fig. 2A,B).

Considering that different tissues exhibit differences in growth regulation, we next examined the adult compound eye to determine the general relationship between TORC2 and dMyc. Similar results were obtained in the eye. Overexpression of dMyc using GMR-Gal4 significantly increased ommatidial size in *lst8*^1^ mutant eyes. Similar size effects were seen when dMyc was overexpressed in the wild-type background (Fig. 2C,D). These data support the hypothesis that dMyc participates in TORC2-driven cell growth. However, as overexpression of dMyc by the Gal4/UAS system causes overgrowth of cells^30^ (Fig. 2C,D), dMyc and TORC2 might also drive cell growth via parallel pathways.

To more precisely assess the interaction between loss of TORC2 and dMyc overexpression, we moderately overexpressed dMyc using the tubulin promoter (*Tub-dmyc*). This construct did not drive cell overgrowth^31^, as wing size and body weight were unaffected by the ubiquitous expression of dMyc. In contrast, growth phenotypes of *dm*^*P0*^ animals were strongly rescued by *Tub-dmyc* (Fig. 2E,F). In *lst8*^1^, *rictor*^*Δ1*^, and *lst8*^1^ *dm*^*P0*^ mutant animals, the reduced body weight and shrunken wings were completely rescued by the *Tub-dmyc* transgene. As a negative control, growth reduction caused by *pink1* depletion was unaffected by dMyc overexpression, suggesting that dMyc only rescues growth defects caused by disruptions in TORC2 signaling. These findings strongly supported of our hypothesis that Myc functions as a downstream effector of TORC2 to affect TORC2-mediated cell growth.

### Loss of Myc function upon disruption of TORC2

**Figure 3:**
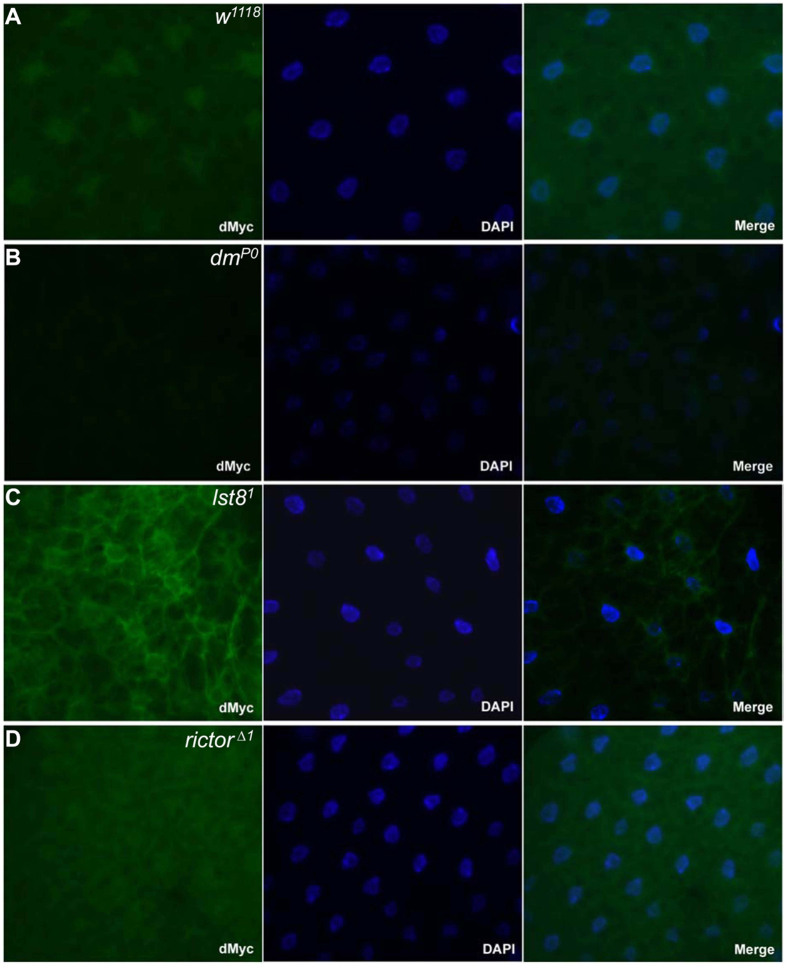
Loss of TORC2 disrupts the nuclear localization of dMyc. **** Immunohistochemistry was used to detect endogenous dMyc protein (green) in larval fat body cells of (**A**) wild-type, (**B**) *dm*^*P0*^, (**C**) *lst8*^1^, and (**D**) *rictor*^*Δ1*^ mutant lava. Hoechst 33342 staining was used to indicate the nucleus (blue).

As a transcription factor, Myc predominantly localizes to the nucleus. As TORC2 might affect cell growth by modulating dMyc pathway, we hypothesized that TORC2 may affect the subcellular localization of dMyc. Immunolabeling experiments were performed using larval fat body tissue to monitor changes in dMyc localization. Antibodies against dMyc revealed that endogenous dMyc localized exclusively to the nucleus in wild-type larval fat body cell, while dMyc staining was dramatically reduced in *dm*^*P0*^ cells (Fig. 3A,B). In fat body tissue lacking LST8 or Rictor, a large portion of dMyc was detected outside of the nucleus, failing to overlap with Dapi (Fig. 3C,D).

**Figure 4:**
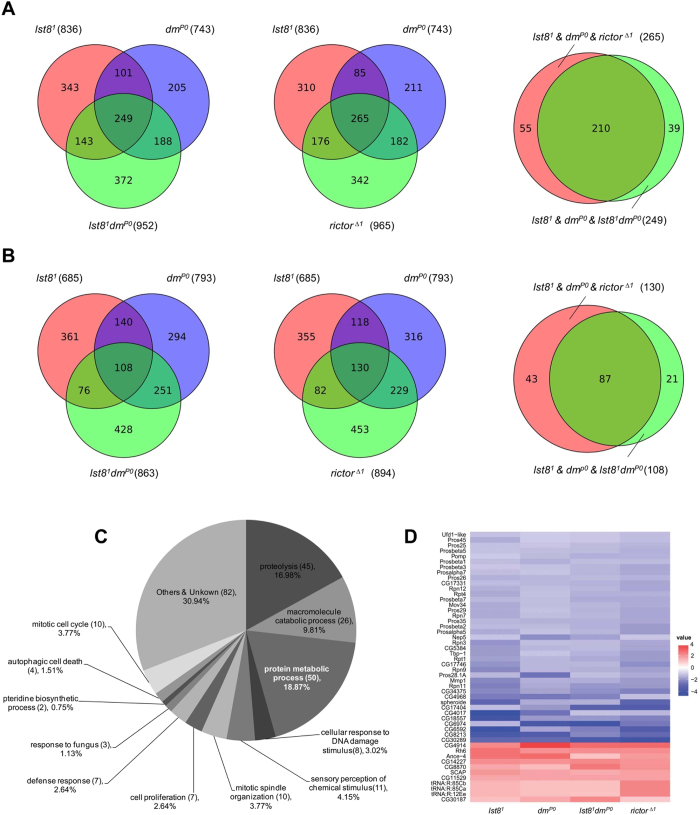
Transcription of dMyc-regulated genes is affected by loss of *lst8*and *rictor*. (**A** and **B**) Venn diagrams showing the overlap of downregulated (**A**) and upregulated (**B**) genes between the four analyzed genotypes: *lst8*, *dmyc*, *lst8 dmyc*, and *rictor*. The number of genes down-regulated and/or up-regulated in all 4 studies is shown in the center of the loden area of the right panels. The overlaps were tested by exact hypergeometric probability (p < 0.001). (**C**) Gene ontology classification of the 265 genes affected by the *lst8*, *rictor*, and *dm* mutations. Counts and percentage in major categories are indicated. (**D**) Heatmap presentation of gene expression for genes in the “protein metabolic process” category. Relative gene expression is encoded in graduated colors from red (upregulated compared with wild type) to blue (downregulated compared with wild type) according to fold change.

As loss of TORC2 disrupted dMyc nuclear localization, we reasoned that TORC2 might also affect the transcriptional activity of dMyc. To test this hypothesis, we used RNA-sequencing (RNA-seq) to compare mRNA levels of wild-type, *lst8*^1^, *dm*^*P0*^, *lst8*^*1*^ *dm*^*P0*^, and *rictor*^*Δ1*^ animals. Large sets of genes were down-regulated in *lst8*^*1*^ (836), *dm*^*P0*^ (743), *lst8*^*1*^ *dm*^*P0*^ (952), and *rictor*^*Δ1*^ (965) tissue (Fig. 4A), indicating transcriptional changes. The number of overlapping genes among the different mutant lines was remarkable. There were 249 overlapping genes among *lst8*^1^, *dm*^*P0*^, and *lst8*^1^ *dm*^*P0*^ tissues, and 265 overlapping genes among *lst8*^1^, *dm*^*P0*^, and *rictor*^*Δ1*^ tissues. Two-hundred and ten genes were downregulated at all 4 mutant conditions (Fig. 4A). Genes were also up-regulated in *lst8*^*1*^ (685), *dm*^*P0*^ (793), *lst8*^1^ *dm*^*P0*^ (862), and *rictor*^*Δ1*^ (894) tissues (Fig. 4B). There were 108 overlapping genes among *lst8*^1^, *dm*^*P0*^, and *lst8*^1^ *dm*^*P0*^ tissues, and 130 overlapping gene among *lst8*^1^, *dm*^*P0*^, and *rictor*^*Δ1*^ tissues. Eighty-seven were up-regulated at all 4 mutant conditions (Fig. 4B).

Gene ontology classification of the 265 overlapping genes among *lst8*^1^, *dm*^*P0*^, and *rictor*^*Δ1*^ are shown in Fig. 4C. Major gene ontology categories included protein metabolic process (18.87%), proteolysis (16.98%), and macromolecule catabolic process (9.81%), which are highly related to cell growth. Together these account for 45.66% of the overlapping genes. We further analyzed genes in the category: “protein metabolic process”. A heat map plot comparing the four genotypes clearly demonstrates the highly similar patterns of expression for genes in this category (Fig. 4D). Transcription levels of these genes were further confirmed with qPCR (Supplementary Fig. S1). We next analyzed genes that overlapping in *lst8*^1^, *lst8*^1^ *dm*^*P0*^, and *rictor*^*Δ1*^ but not in *dm*^*P0*^ mutant tissues. Among these 218 genes, there are a large portion of cell death related genes, indicating that TORC2 has additional functions independent of dMyc in some cellular events such as cell death (Supplementary Fig. S2). Moreover, among 836 genes that are down-regulated in the *lst8*^1^ mutant flies, 101 genes are still down-regulated in the *lst8*^1^ *dm*^*P0*^ mutant flies when comparing with the *dm*^*P0*^ flies (Supplementary Fig. S3). Similarly, among 685 up-regulated genes in the *lst8*^1^ mutant tissues, 71 genes are still up-regulated in the *lst8*^1^ *dm*^*P0*^ double mutant tissues when comparing with the *dm*^*P0*^ tissues (Supplementary Fig. S3). Nevertheless, the highly consistent expression patterns among the 4 mutant stains suggests that TORC2 regulates dMyc transcriptional activity by modulating its nuclear localization.

## Discussion

Here we describe a crosstalk between TORC2 and Myc, two regulators of cell growth. The genetic and cell biology studies in *Drosophila* place TORC2 upstream of MYC in a pathway that regulates cell growth. These results suggest that TORC2 inhibitors may represent effective therapies for treating Myc-driven cancers.

TOR kinase is a highly conserved protein kinase and a central regulator of cell growth. TORC1 has been extensively characterized, but the recent identification of a second TOR complex (TORC2) has complicated the TOR-regulated cell growth pathways. TORC2 regulates growth and metabolism in both mammals and invertebrates^6^,^8^,^9^,^32^. Recent findings indicate that ribosomes physically interact with TORC2 and that this interaction is required for mTORC2 activation. This suggests a critical role for TORC2 in cell growth regulation^33^. However, little is known about the regulation of mTORC2 signaling, or the downstream effectors that implement TORC2-mediated cell growth^4^. Given evidence that TORC2 regulates AKT/FOXO, AKT/FOXO signaling has been considered the major factor acting downstream of TORC2 to control growth. Our findings that AKT- or FOXO does not affect TORC2-mediated cell growth strongly argue against the AKT/FOXO pathway acting downstream of TORC2 in this context^9^.

In the present study, we found that MYC is required for TORC2-regulated cell growth. Mutations in *lst8*, *rictor*, or *dmyc* had similar growth defects, and *lst8 dmyc* double mutants did not have more severe growth phenotypes than *dmyc* mutants. Mosaic analysis in multiple cell types showed that *lst8 dmyc* double mutant cells had similar growth rates and cell sizes as *dmyc* mutant cells within the same animals. Moreover, we established that Myc functions downstream of TORC2 in cell growth. Growth phenotypes associated with loss of TORC2 in the retina, wing, fat body, and the entire body were rescued by the overexpression of dMyc.

Myc protein controls metabolism, cell growth, and proliferation by regulating genes transcribed by RNA Polymerase II, and by stimulating transcription by RNA Polymerases I and III^15^. As Myc is a transcription factor, pathways that regulate the subcellular localization of Myc likely affect its ability to regulate growth and metabolism^34^. Here we found that the lack of TORC2 activity is associated the cytoplasmic accumulation of dMyc. Consequently, many Myc target genes were dysregulated in *lst8* and *rictor* mutant tissues. TORC2-mediated nuclear localization of Myc may represent a novel mechanism by which Myc activity is regulated.

As master regulators of cell growth and metabolism, TORC1 and Myc exhibit coordinated patterns of activity^35^. TORC1 activity is required for cancer cell survival, and TORC1 inhibition has remarkable therapeutic efficacy in Myc-driven hematological cancers^36^. In flies, inhibition of TORC1 by molecular inhibitors, genetic manipulations, or starvation leads to the post-transcriptional downregulation of dMyc followed by the repression of dMyc target genes^16^,^25^. In mammals, it has been reported that TORC1 activity is required for efficient c-MYC translation in TSC2-null Elt3 rat leiomyoma cells^37^, but opposite results have been reported for colorectal cancer cells, in which TORC1 inhibition by rapamycin treatment or knockdown of Raptor results in phosphorylation and accumulation of Myc^38^. Moreover, in *Drosophila* intestinal stem cells, excessive TORC1-driven growth in *TSC* mutants blocked dMyc-induced cell division^39^. These results challenge the notion that TORC1 inhibitors can be used as therapeutic drugs in Myc-driven cancers.

In some cases, such as hyperactivation of AKT signaling, TORC2 is required for proliferation of tumor cells and subsequent tumor growth^33^,^40^. The selective requirement for mTORC2 in tumor development suggests that mTORC2 inhibitors may be of substantial clinical utility^40^. The PI3K/AKT signaling cascade is known to regulate metabolic processes via discrete effectors, such as the TSC (tuberous sclerosis) complex and FOXOs. TORC2 inactivates the FOXO branch without affecting the TSC/TORC1 branch^41^,^42^. It has been demonstrated that FOXO inhibits MYC function to decrease mitochondrial function and to reduce ROS production^43^,^44^. Moreover, a central role for TORC2 in cancer metabolic reprogramming has been proposed, wherein mTORC2 signaling increases cellular c-Myc levels by acetylating FOXO independent of AKT^45^. Here we found that TORC2 controlled cell growth by regulating dMyc via nuclear localization. This regulation of dMyc by TORC2 is likely not through TORC2-mediated inactivation of FOXO, because mutations in *lst8* or *rictor* do not affect dMyc transcription or translation. Moreover, previous reports that TORC2 does not regulate cell growth via AKT/FOXO support the model that TORC2 regulates cell growth via Myc independent of FOXO^9^. This finding suggests that TORC2 inhibitors may represent an effective way of treating Myc-driven cancers.

## Author Contributions

Y.K., H.H. and T.W. designed and performed research; Y.K., H.H., T.C. and T.W. analyzed data; Y.K. and T.W. wrote the paper. All authors approved the final manuscript.

## Additional Information

**How to cite this article**: Kuo, Y. *et al*. Target of Rapamycin Complex 2 regulates cell growth via Myc in *Drosophila*. *Sci. Rep.* **5**, 10339; doi: 10.1038/srep10339 (2015).

## Supplementary Material

Supplementary Information

## References

[b1] Wullschleger S., Loewith R. & Hall M. N. TOR signaling in growth and metabolism. Cell 124, 471–484 (2006). 16469695 10.1016/j.cell.2006.01.016

[b2] Gough N. R. Focus issue: TOR signaling, a tale of two complexes. Sci. Signal 5, eg4 (2012). 22457327 10.1126/scisignal.2003044

[b3] Betz C. & Hall M. N. Where is mTOR and what is it doing there? J. Cell Biol. 203, 563–574 (2013). 24385483 10.1083/jcb.201306041 PMC3840941

[b4] Zoncu R., Efeyan A. & Sabatini D. M. mTOR: from growth signal integration to cancer, diabetes and ageing. Nat. Rev. Mol. Cell Biol. 12, 21–35 (2011). 21157483 10.1038/nrm3025 PMC3390257

[b5] Aimbetov R. *et al.* Integrity of mTORC2 is dependent on the rictor Gly-934 site. Oncogene. 31, 2115–2120 (2012). 21909137 10.1038/onc.2011.404 PMC3305845

[b6] Guertin D. A. *et al.* Ablation in mice of the mTORC components raptor, rictor, or mLST8 reveals that mTORC2 is required for signaling to Akt-FOXO and PKCalpha, but not S6K1. Dev. Cell 11, 859–871 (2006). 17141160 10.1016/j.devcel.2006.10.007

[b7] Hietakangas V., Cohen S. M. & Re-evaluating A. K. T. regulation: role of TOR complex 2 in tissue growth. Genes. Dev. 21, 632–637 (2007). 17369395 10.1101/gad.416307 PMC1820936

[b8] Jones K. T., Greer E. R., Pearce D. & Ashrafi K. Rictor/TORC2 regulates Caenorhabditis elegans fat storage, body size, and development through sgk-1. PLoS biology 7, e60 (2009). 19260765 10.1371/journal.pbio.1000060 PMC2650726

[b9] Wang T., Blumhagen R., Lao U., Kuo Y. & Edgar B. A. LST8 regulates cell growth via target-of-rapamycin complex 2 (TORC2). Mol. Cell Biol. 32, 2203–2213 (2012). 22493059 10.1128/MCB.06474-11 PMC3372270

[b10] Cornu M., Albert V. & Hall M. N. mTOR in aging, metabolism, and cancer. Curr. Opin. Genet. Dev. 23, 53–62 (2013). 23317514 10.1016/j.gde.2012.12.005

[b11] Laplante M., Sabatini D. M. mTOR signaling in growth control and disease. Cell 149, 274–293 (2012). 22500797 10.1016/j.cell.2012.03.017 PMC3331679

[b12] Pearce L. R., Komander D. & Alessi D. R. The nuts and bolts of AGC protein kinases. Nat. Rev. Mol. Cell Biol. 11, 9–22 (2010). 20027184 10.1038/nrm2822

[b13] Jacinto E. *et al.* SIN1/MIP1 maintains rictor-mTOR complex integrity and regulates Akt phosphorylation and substrate specificity. Cell 127, 125–137 (2006). 16962653 10.1016/j.cell.2006.08.033

[b14] Dang C. V. MYC on the path to cancer. Cell 149, 22–35 (2012). 22464321 10.1016/j.cell.2012.03.003 PMC3345192

[b15] Eilers M. & Eisenman R. N. Myc’s broad reach. Genes. Dev. 22, 2755–2766 (2008). 18923074 10.1101/gad.1712408 PMC2751281

[b16] Parisi F. *et al.* Drosophila insulin and target of rapamycin (TOR) pathways regulate GSK3 beta activity to control Myc stability and determine Myc expression *in vivo*. BMC Biol. 9, 65 (2011). 21951762 10.1186/1741-7007-9-65 PMC3235970

[b17] Gallant P. Myc function in Drosophila. Cold Spring Harb. Perspect. Med. 3, a014324 (2013). 24086064 10.1101/cshperspect.a014324 PMC3784813

[b18] Orian A. *et al.* Genomic binding by the Drosophila Myc, Max, Mad/Mnt transcription factor network. Genes. Dev. 17, 1101–1114 (2003). 12695332 10.1101/gad.1066903 PMC196053

[b19] Hulf T. *et al.* Whole-genome analysis reveals a strong positional bias of conserved dMyc-dependent E-boxes. Mol. Cell Biol. 25, 3401–3410 (2005). 15831447 10.1128/MCB.25.9.3401-3410.2005 PMC1084277

[b20] Grewal S. S., Li L., Orian A., Eisenman R. N. & Edgar B. A. Myc-dependent regulation of ribosomal RNA synthesis during Drosophila development. Nat. Cell Biol. 7, 295–302 (2005). 15723055 10.1038/ncb1223

[b21] Marshall L., Rideout E. J. & Grewal S. S. Nutrient/TOR-dependent regulation of RNA polymerase III controls tissue and organismal growth in Drosophila. EMBO J. 31, 1916–1930 (2012). 22367393 10.1038/emboj.2012.33 PMC3343326

[b22] Iritani B. M. & Eisenman R. N. c-Myc enhances protein synthesis and cell size during B lymphocyte development. Proc. Natl. Acad. Sci. USA 96, 13180–13185 (1999). 10557294 10.1073/pnas.96.23.13180 PMC23921

[b23] Johnston L. A., Prober D. A., Edgar B. A., Eisenman R. N. & Gallant P. Drosophila myc regulates cellular growth during development. Cell 98, 779–790 (1999). 10499795 10.1016/s0092-8674(00)81512-3 PMC10176494

[b24] Conacci-Sorrell M., McFerrin L. & Eisenman R. N. An overview of MYC and its interactome. Cold Spring Harb. Perspect. Med. 4, a014357 (2014). 24384812 10.1101/cshperspect.a014357 PMC3869278

[b25] Teleman A. A., Hietakangas V., Sayadian A. C. & Cohen S. M. Nutritional control of protein biosynthetic capacity by insulin via Myc in Drosophila. Cell Metab. 7, 21–32 (2008). 18177722 10.1016/j.cmet.2007.11.010

[b26] Hsu P. P. *et al.* The mTOR-regulated phosphoproteome reveals a mechanism of mTORC1-mediated inhibition of growth factor signaling. Science 332, 1317–1322 (2011). 21659604 10.1126/science.1199498 PMC3177140

[b27] Trapnell C., Pachter L. & Salzberg S. L. TopHat: discovering splice junctions with RNA-Seq. Bioinformatics 25, 1105–1111 (2009). 19289445 10.1093/bioinformatics/btp120 PMC2672628

[b28] Trapnell C. *et al.* Transcript assembly and quantification by RNA-Seq reveals unannotated transcripts and isoform switching during cell differentiation. Nat. Biotechnol. 28, 511–515 (2010). 20436464 10.1038/nbt.1621 PMC3146043

[b29] Huang da W., Sherman B.T. & Lempicki R.A. Systematic and integrative analysis of large gene lists using DAVID bioinformatics resources. Nat. Protoc. 4, 44–57 (2009). 19131956 10.1038/nprot.2008.211

[b30] Secombe J., Li L., Carlos L. & Eisenman R. N. The Trithorax group protein Lid is a trimethyl histone H3K4 demethylase required for dMyc-induced cell growth. Genes. Dev. 21, 537–551 (2007). 17311883 10.1101/gad.1523007 PMC1820896

[b31] de la Cova C., Abril M., Bellosta P., Gallant P. & Johnston L. A. Drosophila myc regulates organ size by inducing cell competition. Cell 117, 107–116 (2004). 15066286 10.1016/s0092-8674(04)00214-4

[b32] Soukas A. A., Kane E. A., Carr C. E., Melo J. A. & Ruvkun G. Rictor/TORC2 regulates fat metabolism, feeding, growth, and life span in Caenorhabditis elegans. Genes. Dev. 23, 496–511 (2009). 19240135 10.1101/gad.1775409 PMC2648650

[b33] Zinzalla V., Stracka D., Oppliger W. & Hall M. N. Activation of mTORC2 by association with the ribosome. Cell 144, 757–768 (2011). 21376236 10.1016/j.cell.2011.02.014

[b34] Li Z. & Hann S. R. Nucleophosmin is essential for c-Myc nucleolar localization and c-Myc-mediated rDNA transcription. Oncogene. 32, 1988–1994 (2013). 22665062 10.1038/onc.2012.227 PMC3855075

[b35] Chan J. C. *et al.* AKT promotes rRNA synthesis and cooperates with c-MYC to stimulate ribosome biogenesis in cancer. Sci. Signal 4, ra56 (2011). 21878679 10.1126/scisignal.2001754

[b36] Pourdehnad M. *et al.* Myc and mTOR converge on a common node in protein synthesis control that confers synthetic lethality in Myc-driven cancers. Proc. Natl. Acad. Sci. USA 110, 11988–11993 (2013). 23803853 10.1073/pnas.1310230110 PMC3718086

[b37] Babcock J. T. *et al.* Mammalian target of rapamycin complex 1 (mTORC1) enhances bortezomib-induced death in tuberous sclerosis complex (TSC)-null cells by a c-MYC-dependent induction of the unfolded protein response. J. Biol. Chem. 288, 15687–15698 (2013). 23612979 10.1074/jbc.M112.431056 PMC3668728

[b38] Tan J. *et al.* B55beta-associated PP2A complex controls PDK1-directed myc signaling and modulates rapamycin sensitivity in colorectal cancer. Cancer Cell 18, 459–471 (2010). 21075311 10.1016/j.ccr.2010.10.021

[b39] Amcheslavsky A., Ito N., Jiang J. & Ip Y. T. Tuberous sclerosis complex and Myc coordinate the growth and division of Drosophila intestinal stem cells. J. Cell Biol. 193, 695–710 (2011). 21555458 10.1083/jcb.201103018 PMC3166862

[b40] Guertin D. A. *et al.* mTOR complex 2 is required for the development of prostate cancer induced by Pten loss in mice. Cancer Cell 15, 148–159 (2009). 19185849 10.1016/j.ccr.2008.12.017 PMC2701381

[b41] Fu Z. & Tindall D. J. FOXOs, cancer and regulation of apoptosis. Oncogene. 27, 2312–2319 (2008). 18391973 10.1038/onc.2008.24 PMC2819403

[b42] Oh W. J. & Jacinto E. mTOR complex 2 signaling and functions. Cell Cycle 10, 2305–2316 (2011). 21670596 10.4161/cc.10.14.16586 PMC3322468

[b43] Jensen K. S. *et al.* FoxO3A promotes metabolic adaptation to hypoxia by antagonizing Myc function. EMBO J. 30, 4554–4570 (2011). 21915097 10.1038/emboj.2011.323 PMC3243591

[b44] Ferber E. C. *et al.* FOXO3a regulates reactive oxygen metabolism by inhibiting mitochondrial gene expression. Cell Death Differ 19, 968–979 (2012). 22139133 10.1038/cdd.2011.179 PMC3354049

[b45] Masui K. *et al.* mTOR complex 2 controls glycolytic metabolism in glioblastoma through FoxO acetylation and upregulation of c-Myc. Cell Metab. 18, 726–739 (2013). 24140020 10.1016/j.cmet.2013.09.013 PMC3840163

