# Supplementary material for: Target of Rapamycin Complex 2 regulates cell growth via Myc in Drosophila
Source: Sci Rep. 2015 May 22;5:10339. doi: 10.1038/srep10339 (PMC4441130; doi:10.1038/srep10339)
Supplement: Supplementary Information [file srep10339-s1.pdf]

Target of Rapamycin Complex 2 regulates cell growth via Myc in *Drosophila*

Ying Kuo, Huanwei Huang, Tao Cai, and Tao Wang

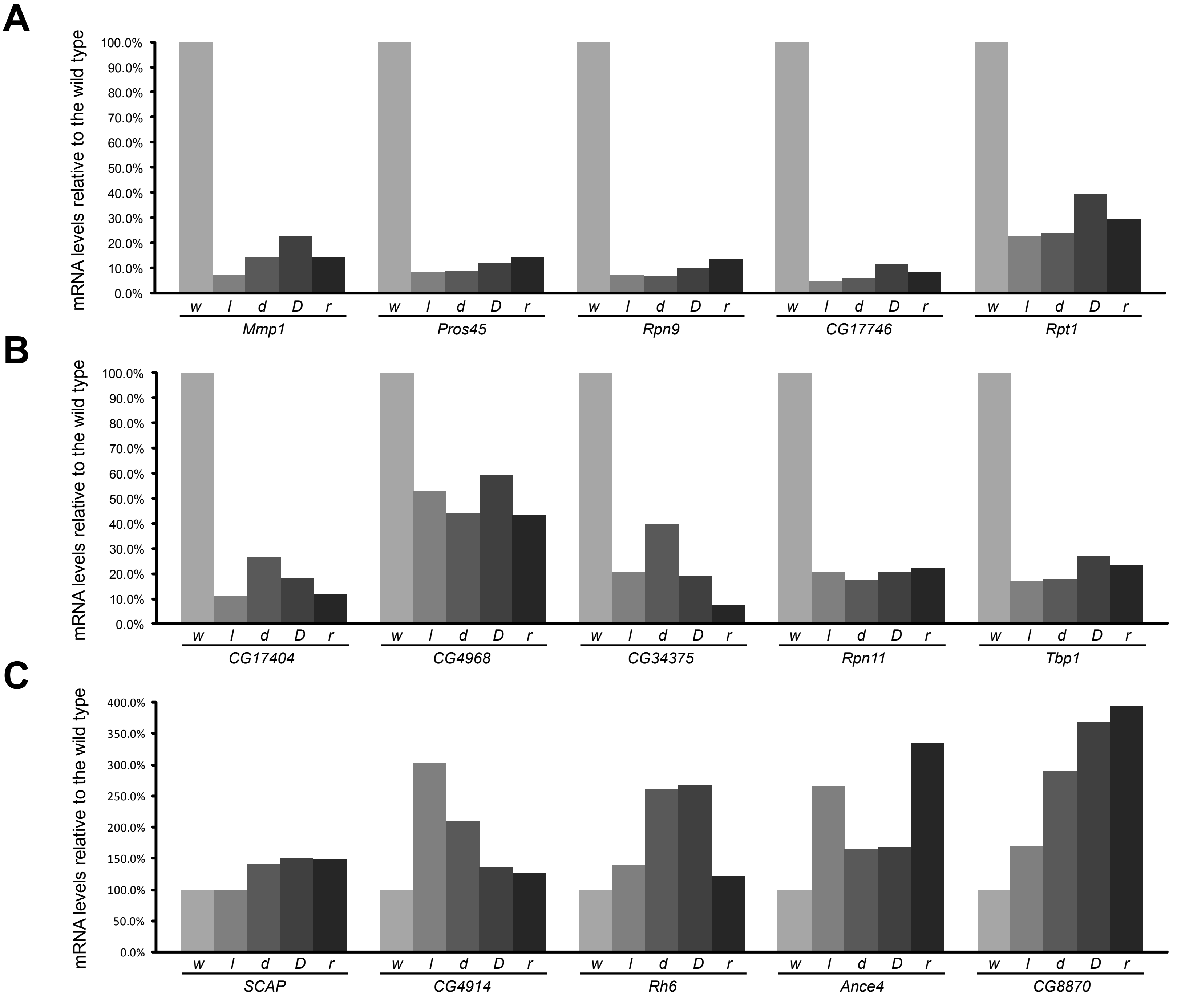

Supplementary Figure S1. Confirmation of RNA-seq results by Quantitative PCR. (A-C) Quantitative PCR of selected gene expression identified from the comparison of RNA-seq results. Total cDNA and mRNA were extracted and inversed from flies with genotypes of wild-type control (*w*), *lst8*^{1} (*l*), *dm*^{P0} (*d*), *lst8*^{1} *dm*^{P0} (*D*) and *rictor*$^{\Delta 1}$ (*r*).

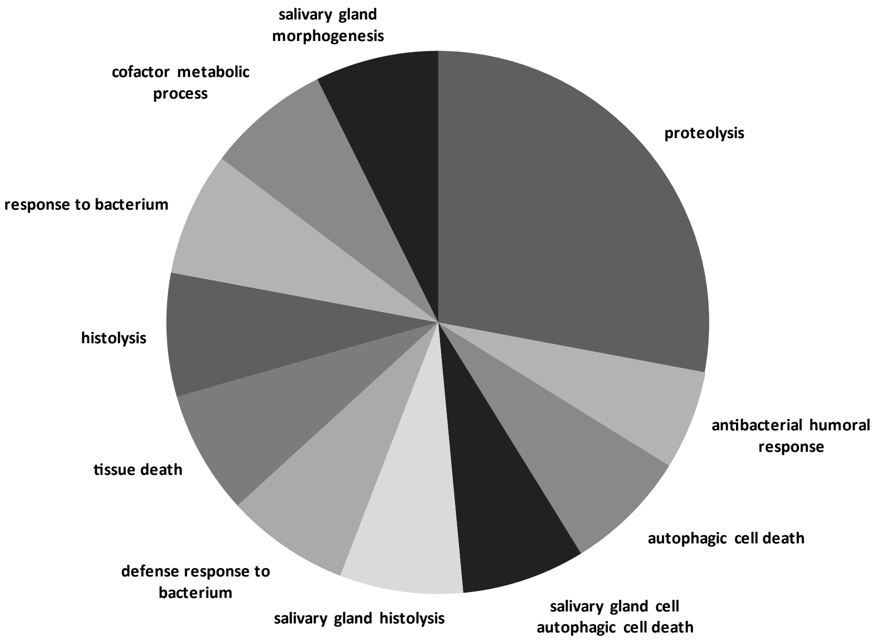

Supplementary Figure S2. Gene ontology classification of the 214 genes affected by the *lst8*^{1} and *lst8*^{1} *dm*^{P0} mutations but not by *dm*^{P0} mutations.

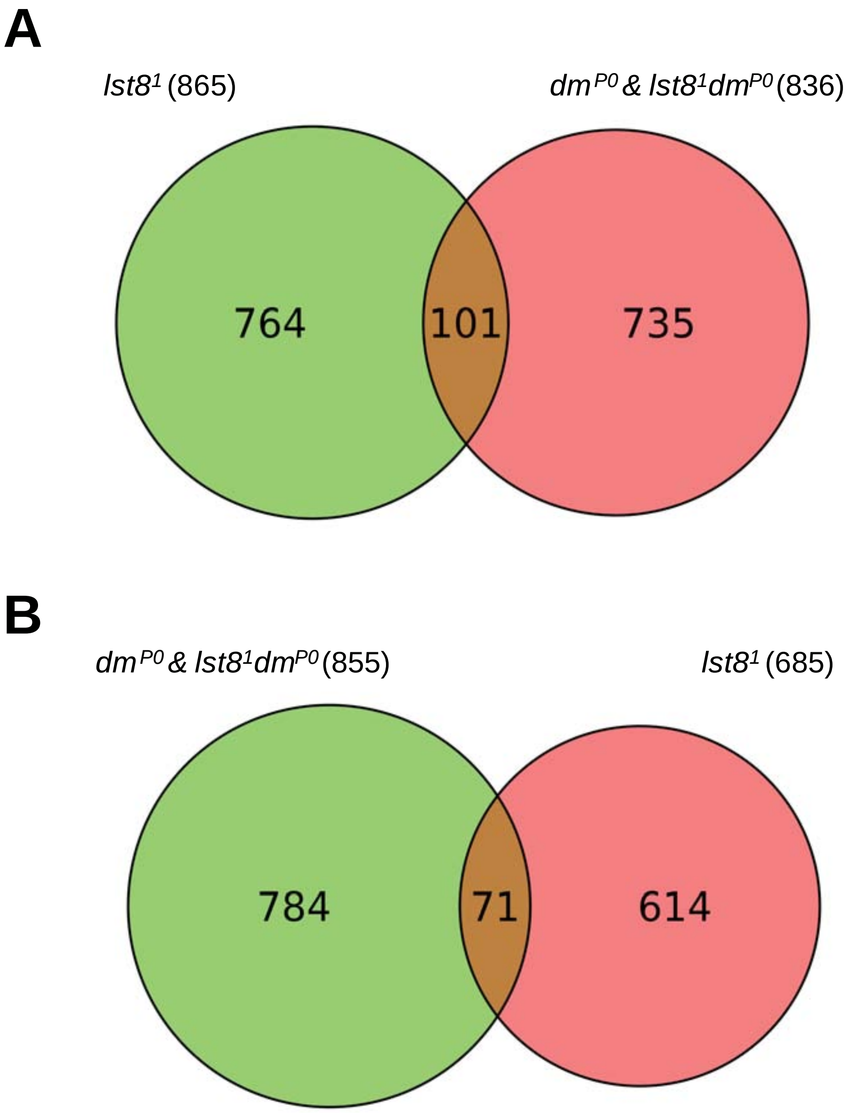

Supplementary Figure S3. Venn diagrams comparing of downregulated (A) and upregulated (B) genes between *lst8*^{1} versus control and *lst8*^{1} *dm*^{P0} versus *dm*^{P0}.
